# Housing insecurity among black women surviving intimate partner violence during the COVID-19 pandemic: an intersectional qualitative approach

**DOI:** 10.1186/s12889-024-17965-5

**Published:** 2024-02-16

**Authors:** Tiara C. Willie, Sabriya L. Linton, Shannon Whittaker, Karlye A. Phillips, Deja Knight, Mya C. Gray, Gretta Gardner, Nicole M. Overstreet

**Affiliations:** 1 Department of Mental Health, Johns Hopkins Bloomberg School of Public Health, 624 N. Broadway, 21205 Baltimore, MD USA; 2 Department of Social and Behavioral Sciences, Yale University School of Public Health, New Haven, CT USA; 3 Department of International Health, Johns Hopkins Bloomberg School of Public Health, Baltimore, MD USA; 4 School of Law, North Carolina Central University, Durham, NC USA; 5 Ujima, The National Center on Violence Against Women in the Black Community, Washington, DC USA; 6 Department of Psychology, Clark University, Worchester, MA USA

**Keywords:** Black women, Intimate partner violence, Housing, Eviction, COVID-19

## Abstract

**Background:**

Housing instability is highly prevalent among intimate partner violence (IPV) survivors, and the coupling consequences of structural racism, sexism, classism, and the COVID-19 pandemic, may create more barriers to safe and adequate housing, specifically for Black women IPV survivors. In particular, the consequences of the COVID-19 pandemic had the potential to amplify disadvantages for Black women IPV survivors, yet very little research has acknowledged it. Therefore, the current study sought to assess the experiences of housing insecurity among Black women experiencing intimate partner violence (IPV) while navigating racism, sexism, and classism during the COVID-19 pandemic.

**Methods:**

From January to April 2021, we conducted in-depth interviews with 50 Black women experiencing IPV in the United States. Guided by intersectionality, a hybrid thematic and interpretive phenomenological analytic approach was used to identify sociostructural factors shaping housing insecurity.

**Results:**

Our findings demonstrate the various ways in which the COVID-19 pandemic shaped Black women IPV survivors’ ability to obtain and sustain safe housing. We derived five themes to capture factors contributing to housing experiences: challenges with separate and unequal neighborhoods; pandemic-related economic inequalities; economic abuse limitations; and strategies to maintain housing.

**Conclusions:**

Obtaining and maintaining safe housing during the COVID-19 pandemic was difficult for Black women IPV survivors who were also navigating racism, sexism, and socioeconomic position. Interventions are needed to reduce the impact of these intersecting systems of oppression and power to facilitate the resources necessary for Black women IPV survivors to identify safe housing.

**Supplementary Information:**

The online version contains supplementary material available at 10.1186/s12889-024-17965-5.

## Background

In the U.S., the primary and secondary consequences of the COVID-19 pandemic placed Black women at elevated risk for adverse mental and behavioral health [1, 2]. While policies such as the Stay-At-Home order allowed many to work from home, essential workers were required to work outside of their home, which placed them at increased risk of contracting COVID-19 virus [3] Historically, occupation segregation, driven by structural racism and sexism, contributed to the overrepresentation of Black women employed in essential work and low-wage occupations (e.g., childcare) [4, 5]. Consequently, Black women employed in essential work and their families were at disproportionate risk of COVID-19 exposure [5]. Black women had a higher mortality rate due to COVID-19 compared to white women and men [6], and experienced the highest unemployment rate during the COVID-19 pandemic [7, 8]. The economic vulnerabilities that Black women endured were imposed by structural determinants, exacerbated during the COVID-19 pandemic, and situated women at greater risk for intimate partner violence (IPV).

Before the COVID-19 pandemic, Black women reported some of the highest IPV prevalence nationally [9], and IPV prevalence increased during the pandemic [10, 11] Tensions between intimate partners regarding work-life conflicts and economics during the pandemic may have disrupted relationship dynamics and put women at risk for IPV [11, 12]. Particularly, experiencing job loss during the pandemic may have led to fewer resources, created additional financial strain in a relationship, and this may have manifested into relationship conflicts [11]. Additionally, having an essential job requires in-person activities, and leads to greater risk of viral exposure for the employee and their family, which could result in conflicts if intimate partners misalign on the “cost” of this risk.

### Pandemic-related economic crises and partner violence

Contextualizing the landscape of IPV and economics for Black women during the pandemic is particularly important. Specifically, the wage gap demonstrates the devaluation of U.S. Black women’s work as women are severely underpaid [13]; additionally, 75% of Black mothers are primary earners [13] Navigating these economic vulnerabilities during the pandemic could have exacerbated the risk of IPV for Black women and their families.

The socioeconomic ramifications of the COVID-19 pandemic increased housing insecurity, which may impede access to safe housing for Black women surviving IPV. Intimate partners who use economic abuse may misappropriate finances and damage credit [14], which further places survivors’ housing in jeopardy. The impacts of economic abuse may be more pronounced due to the increased economic vulnerabilities superimposed by the pandemic. In addition to strain of COVID-19 leading to more relationship conflicts, stay-at-home orders and social distancing policies also forced IPV survivors to be “housed” with abusive partners, and isolated from informal social support [11]. Some survivors had few options for safe housing and were forced to stay in abusive relationships during the pandemic [15]. Furthermore, Black IPV survivors may have experienced rental discrimination due to racialized gendered stereotypes of Black womanhood and property owners’ efforts to prevent “nuisance” citations [16].

### Employing an intersectional framework

Maintaining safe adequate housing was a public health concern during the COVID-19 pandemic, however, very little is known about how Black women experiencing IPV at diverse intersectional positions navigated the housing crisis and how these experiences are linked to interlocking systems of privilege and power (e.g., racism, sexism). Intersectionality is a social justice-oriented theoretical framework, which posits that multiple intersecting identities at the individual-level can reflect interlocking systems of privilege and power [17, 18] This framework addresses the socio-political identity of marginalized populations by drawing upon concepts of Black Feminist Theory and Critical Race Theory. Position and power, two constructs of intersectionality, underpin the foundation for IPV survivorship and perpetration [17, 19, 20]. This theory shapes best provides context to the integrative systems of power that Black women survivors of IPV experience. Black women experiencing IPV with social identities of race, gender, and homeownership status reflect macro-level systems of racism, sexism, and socioeconomic position (SEP). While emerging research has examined the impacts of the pandemic on IPV incidence and housing insecurity separately, very little attention has been given to Black women who are situated at multiple axes of identity and oppression. Therefore, this qualitative study sought to describe Black women’s experiences finding, obtaining, and maintaining safe housing during the COVID-19 pandemic while navigating sociostructural challenges.

## Methods

From January through June 2021, Black women IPV survivors were recruited through flyers disseminated by domestic violence agencies and social media to participate in a qualitative interview. Flyers stated that this was a study on Black women’s experiences with relationship conflicts and housing during the pandemic. Inclusion criteria included: self-identified as Black cisgender female; resided in the U.S.; reported ≥ 1 form of IPV within the past six months (i.e., physical, sexual, psychological); and ≥ 18 years. Interviews were conducted to elicit personal narratives of housing during COVID-19. Black women IPV survivors completed a one-time virtual, semi-structured interview. Prior to the start of the interview, the interviewer obtained verbal consent from the participant. The semi-structured interview guide contained questions regarding pandemic impacts on housing and interpersonal relationships (**Supplementary File 1**). The interview guide included questions on housing status and changes to housing status during COVID-19. At the end of the interview, Housing Timeline Follow Back calendar and demographic questions were asked (i.e., sexual orientation and relationship status). Analytical memos were created after each interview, and the interviewers debriefed weekly. Interviews were audio-recorded, transcribed verbatim, and lasted on average 60 min. Participants were remunerated $45. All participants provided oral consent. The Johns Hopkins Bloomberg School of Public Health IRB (#14192) approved all study procedures.

Interviews were conducted, coded, and analyzed by Black cisgender women. Interviews were analyzed using a hybrid thematic and interpretative phenomenological approaches [21]. Thematic analysis requires looking across the data to identify themes [22]. Interpretative phenomenological analysis focuses on understanding the experience of the “participant in context” and prioritizes their experience [22] Five coders read and coded the same two initial transcripts. After coding each transcript, comparison of coded segments and discussion of memos were used to develop the codebook iteratively. Any discrepancy in initial coding was discussed among team members until a consensus was reached. The codebook contained deductive codes based on prior literature on IPV and housing [15, 16, 23, 24] Inductive codes were also identified. The codebook was used by the five coder team to line-by-line code the remaining interviews. No new codes were added to the codebook after codebook development. Weekly meetings were held to discuss inconsistencies in code application and discuss prior assumptions that shaped interpretation. All transcripts were coded in Dedoose Version 4.5 [25].. The PI (also a coder and interviewer) reviewed all excerpts under the Housing parent code and categorized excerpts to create superordinate themes describing participants’ lived experiences. Specifically, in a matrix form, interpretations of subordinate codes (e.g., partner sabotaged credit) related to housing were group based on patterns across SEP. The patterns were later categorized into second-order or superordinate themes (e.g., experiences of economic abuse derailed stable housing). An audit trail was maintained with memos and these memos were discussed in team meetings. The PI sought feedback on the resulting themes from a domestic violence advocate, housing scientist, and intersectionality scientist whom all identified as Black women. Pseudonyms were used for participant’s confidentiality.

## Results

Table 1 summarizes characteristics for 50 Black women participants. The average age was 35 years with a range of 18–67 years. Half of the participants (50%) resided in the Southwest, followed by the Southeast region (34%), Northeast region (8%), West (4%) and Midwest regions (4%). Most participants identified as heterosexual (88%), followed by lesbian or bisexual (4%), and asexual (2%). Half of the participants (50%) reported physical, psychological, and sexual IPV experiences in the past year. Nearly three-fifths of the participants (58%) reported renting their homes at the time of the interview, followed by being homeless (14%), being a homeowner (10%), residing in a domestic violence shelter (10%), and residing in subsidized housing (8%).

**Table 1:** Characteristics of Black Women Who Participated in Semi-Structured Interviews, January 2021 and April 2021

	N (%)
**Overall**	50 (100.0)
**Age**, *mean (range)*	35.6 (18–67)
**Age** in years	
Less than 26 years old	7 (14)
Greater than or equal to 26 years old	43 (86)
**Marital status**	
Never married	20 (40)
Married	13 (26)
Divorced or separated	17 (34)
**Region**	
West	2 (4)
Southwest	25 (50)
Midwest	2 (4)
Southeast	17 (34)
Northeast	4 (8)
**Sexual Orientation**	
Heterosexual	44 (88)
Lesbian or Bisexual	2 (4)
Asexual	1 (2)
Did not disclose	3 (6)
**Past-year Intimate Partner Violence**	
Psychological only	9 (18)
Sexual and Psychological	5 (10)
Physical and Psychological	11 (22)
Physical, Psychological, and Sexual	25 (50)
**Place of Residence**	
Renter	29 (58)
Homeowner	5 (10)
Subsidized housing	4 (8)
Homeless	7 (14)
Domestic Violence Shelter	5 (10)

Five themes demonstrate Black women IPV survivors’ housing experiences during the pandemic: *challenges with separate and unequal neighborhoods; pandemic-related economic inequalities; economic abuse as a factor limiting housing options; and strategies to maintain housing during the pandemic*. We discuss how homeownership status (i.e., homeowner, renter, unstable/no housing), as indicative of their SEP, may shape their experiences. Table 2 contains additional quotes for each theme.

**Table 2:** Themes and Additional Illustrative Quotes: Black Women Experiencing IPV in United States, 2021

Themes	Additional Illustrative Quotes
**Pandemic-related economic inequalities**	“I was saying we had to move to a cheaper house. We had to also prioritize on most of the various things that could be probably having. We had to live a cheaper lifestyle.” (Ashley, 26 + years old, Washington, Living with Family) “Initially, we come to work, so we’re gonna go ahead and complete that task and just stay and find work, find housing. Now, we are considered in homeless transition from hotel to hotel as far as that goes. We’ve been calling for assistance.” (Essence, 26 + years old, Georgia, Homeless)
**Economic abuse**	“I would say when it came to my job. I’ve had to quit at least two jobs because he would find me, and my bosses would kind of feel uneasy about me working there. That makes people feel really uneasy when someone’s calling and threatening to just show up and hurt people or try to hurt you.” (Danika, < 26 years old, North Carolina, Apartment Renter)
**Strategies to maintain housing**	“All my friends know this. I used to have a food budget before. Now, I just buy the minimal stuff. Everybody is saying, “Oh, my goodness. You’ve lost so much weight.” Really? That’s because I’m trying to make the rent. I’m not spending like that. Maybe breakfast is just oatmeal. Maybe lunch is just a bread slice and some chicken, and dinner maybe would be rice and veggies, just really minimal. I’m trying to put that money towards the rent.” (Alice, 26 + years old, District of Columbia, Apartment Renter) “I think so many companies had to laid off their employees because there was no money to pay their salaries. My company was one of the companies who did do that. I tried to talk to my landlord because now I had exhausted all my savings. I had two months’ debt for rent, so the landlord was tired and got me evicted. I had no shelter.” (Gina, 26 + years old, Texas, Homeless)

### Challenges with separate and unequal neighborhoods

Throughout their interviews, Black women IPV survivors discussed how experiencing racial residential segregation and gentrification shaped their housing. While racial residential segregation and gentrification predates COVID-19, these structural conditions interfered with Black women survivors’ ability to obtain and maintain safe, adequate housing during the pandemic.

A few IPV survivor renters noted a housing shortage which was inextricably linked to an ongoing legacy of racialized housing practices and residential segregation. Aaliyah said, “I feel like there’s not enough [housing] especially here in [Town]. It’s not enough housing and not enough places to stay for Black people. It’s like they put us all in one little area, and then it’s the bad area.” (26 + years old, Illinois, Apartment Renter) Black women IPV survivors were searching for safe rentals amid a national housing shortage; however, this search is exacerbated by legacies of housing discrimination which disenfranchised Black Americans by devaluing Blackness and removed them from systems of investment.

Several women homeowners in this study discussed how racial residential segregation creates enclaves of concentrated poverty, disinvestment, and neighborhood deprivation. As institutions intentionally divest from segregated communities, these communities may disproportionately have poor neighborhood infrastructure and a lack of resources such as adequate schools and employment opportunities; all of which exacerbate local economic disparities. For example, Benita shared,

“I live in [County]. We have a Black mayor and a Black sheriff…but I noticed when I look at my [County] versus [County]; those counties are actually white, they have more resources, and they know about them. I’m always looking up [resources], and I was not able to find them. I had a conversation with one of the directors, and he mentioned how there is a racial disparity in terms of the funding, and in terms of the funding our County gets versus [County] will get.” (26 + years old, Georgia, Homeowner).

According to this homeowner, her community, with Black leadership, was enduring divestment than neighboring counties with more white residents. This homeowner recognized that white counties received more resources than Black counties due to race. This racial disparity may leave residents feeling excluded from attaining the necessary resources needed to support themselves and their networks.

Black women homeowners, especially those residing in racially segregated communities, were also dealing with the consequences of gentrification like police-perpetrated discrimination. For example, Chantel reflected on how gentrification has changed her ability to mobilize in her home and neighborhood:

“I feel like I’ve been traumatized multiple times and been stopped by the police inside of my complex because I have locks in my hair. I feel like I’m being picked on. I feel like we’re being picked on. That’s one of the things when it comes to policing in communities like mine, especially because we’re being gentrified.” (26 + years old, Washington, DC, Homeowner).

Using an intersectionality lens, the *Challenges with separate and unequal neighborhoods* theme captures how racism and classism intersect to shape women’s access to adequate housing. Specifically, women’s narratives demonstrate how present-day challenges of disinvestment and police-perpetrated discrimination in women’s neighborhoods are consequences of historical racialized policies and practices (i.e., mechanisms of racism and classism).

### Pandemic-related economic inequalities disrupted housing stability

Throughout their narratives, several Black women IPV survivors described experiencing housing insecurity, in large part, due to the negative economic impacts of the pandemic on their employment. Survivors often discussed being in one of two socioeconomically vulnerable positions during the pandemic: unemployed and underemployed. Dionne describes underemployment as:

“I managed because I had 36 or 40 hours a week, a decent paycheck. But after COVID, they started laying people off. Some weeks I got about one or two days [of work] within a two-week pay period.” (26 + years old, Texas, Subsidized Renter).

Furthermore, many participants discussed how underemployment had a cumulative impact on women’s salary which resulted in delayed payments for housing. For example, Ericka stated:

“When you are getting the pay cut—it just did not become a pay cut, but it also became delayed salary, especially when the pandemic hit us. So the delay in salary would also bring delay in paying of the rent.” (< 26 years old, New York, Apartment Renter).

Similarly, a few parenting Black women survivors often found themselves moving multiple times with their children during the pandemic due to underemployment. For instance, Faith stated:

“We’ve moved two times since COVID because of financial reasons like I’m not making the money I used to make. I cannot get a full check because I always have to leave. I can only work between the hours of the kids at school. Then, either I’m late, or I have to leave early so that I can get them. I cannot afford childcare and pay bills.” (Texas, Apartment Renter).

The *Pandemic-related economic inequalities* theme underscores that the consequences of the pandemic exacerbated existing systems of racism, sexism, and classism that disadvantaged Black women survivor’s economic independence and amplified housing insecurity. In particular, the historical impacts of racism, sexism, and classism have operated through discriminatory practices in high-wage employment, resulting in an overrepresentation of Black women in essential industries (i.e., childcare, grocery) [4, 5]. While this industry was necessary during the pandemic, Black women still experienced under- and unemployment during the lockdown orders. The intersections of racism, sexism, and classism disrupted Black women survivor’s ability to finances, which increased women’s concerns of housing stability.

### Economic abuse further restricts housing options

Several Black women participants discussed economic abuse being perpetrated by their romantic partners and its long-term implications for their housing. For example, abusive partners would restrict their economic independence: “I was supposed to stay in the house, so I did not have the freedom of going out to look for a job.” (Grace, 26 + years old, New York, Homeless) Coercing women into unemployment during the pandemic pressures Black women IPV survivors to become more dependent on their partners to provide necessities (e.g., food, shelter). Black women IPV survivors who become economically dependent on their partners during the pandemic may have limited finances to leave their relationship and find alternative safe housing.

Partners damaging survivors’ financial history is another form of economic abuse that limited women’s housing options. For example, Harmony noted “He took out a credit card in my name and ran it up.” (< 26 years old, North Carolina, Apartment Renter) A survivors’ credit history is likely reviewed when applying for certain utilities, housing, and employment [26]. During the pandemic, Black women IPV survivors may have been unable to relocate to safer areas due to the inherent consequences of economic abuse on their credit histories, and poor credit health may further impact their abilities to secure safe and affordable housing in the future.

Similarly, most women shared how partners perpetrating economic abuse can intentionally misappropriate housing funds. Ericka shared, “My husband would fail to pay the bills in terms of the rent and so we faced getting evicted.” (< 26 years old, New York, Apartment Renter) Economically abusive partners’ inability to pay the housing-related bills puts survivors at elevated risk of eviction during the pandemic. Some participants voiced that economically abusive behaviors could also result in increased risk for incarceration due to unpaid utility bills. Iyana shared:

“I told my caseworker, “I do not want to be stuck in this lease or break my lease,” because I’m trying to clear my credit because he messed my credit up…He had a water bill in my name, a power bill, and a gas bill…I got two citations: one for tampering with water distribution and another one for the bill not being paid. I did not know. They said that I have a warrant for my arrest. I said, “For what? Because I did not do anything. I do not commit crimes.” (26 + years old, Texas, Domestic Violence Housing Program).

An intersectionality perspective of the *Economic abuse* theme allows a deeper investigation of how partner-perpetrated economic abuse and unequal power dynamics in romantic partnerships uniquely impact Black women IPV survivors’ housing. Specifically, these narratives demonstrate the ways in which economic abuse shapes Black women IPV survivors and how the ongoing, compounding effects of racism, sexism, and classism such as occupational segregation and racialized housing practices, drastically reduce Black women’s access to affordable housing.

### Strategies to maintain housing

The economic repercussions of the COVID-19 pandemic necessitated that Black women IPV survivors used various survival strategies to maintain their housing. Interviews with several Black women IPV survivors indicated that strategic resource tradeoffs were made to sustain housing during the pandemic. For example, Madison noted: “Yeah, I had to pawn my TV. I had to pawn my title to my car.” (26 + years old, Texas, Apartment Renter) Participants also sacrificed other physical needs. Nia shares: “Either it’s [rent] going to be late, or I have to go without something. We might need groceries, but we’ve got to pay our rent.” (26 + years old, Georgia, Apartment Renter).

In response to the eviction filings, most participants tried to identify eviction moratoriums to help keep their housing intact, but there was confusion about the eviction moratoriums. For example, Olivia shared: “They cut my hours in half, and then I lost my work. I had to wait for unemployment. Every fourth of the month, there was an eviction notice. ‘We’re filing in eviction court.’ People tell me that’s not real and that they did not actually do that because of the government, but I don’t know what is actually true. I just know the letters that I receive.” (26 + years old, Maryland, Apartment Renter).

Women homeowners also experienced tradeoffs but those tradeoffs involved assets and household employees. Raven discussed liquidating her assets during the pandemic: “I had to pretty much sell my whole house. I sold everything from furniture to clothing just to make ends meet.” (26 + years old, Homeowner, Georgia) In response to managing housing expenses, Shani mentioned laying off a household employee: “I had to forego living with a nanny because I was not working.” (< 26 years old, Homeowner, South Carolina).

## Discussion

The intersectional invisibility of Black women IPV survivors is apparent in extant research addressing housing, COVID-19, and IPV. Extant research has failed to consider how systems of power and oppression can produce housing obstacles unique to Black women surviving IPV during a pandemic. Black women in this study recounted how the pandemic exacerbated and created difficulties with their housing stability. Guided by Intersectionality [17, 18], our findings underscore how racism, sexism, and SEP shaped women’s housing experiences across diverse homeownership statuses.

### Societal- and community-level determinants

In this study, racial residential segregation was a key obstacle for Black women IPV survivors overall; however, women homeowners’ narratives emphasized the impacts of gentrification. Black women described how racialized housing practices constructed racially segregated areas, which have endured disinvestment and a lack of resources. Some Black women IPV survivors who were renting homes were residing in racially segregated neighborhoods with limited access to resources needed to flourish. Racialized housing practices also impede Black women IPV survivors’ ability to leave their relationship and find housing in non-segregated neighborhoods with more resources. Moreover, Black women IPV survivors who were homeowners experienced hyperpolicing and discrimination in gentrifying communities. Hyperpolicing may occur in gentrifying communities to protect the interests of middle-class white people, and real estate assets [27]. Survivors’ experience of gentrification and policing aligns with the postindustrial policing hypothesis. The postindustrial policing hypothesis postulates that policing strategies becomes more aggressive and targets populations (e.g., people of color) when communities are being gentrified [28, 29]. Black women IPV survivors who are homeowners are trying to maintain safe housing but police-perpetrated discrimination and IPV undermine their safety.

Consequently, these demographic and real estate changes in gentrifying communities could increase the incidence of discrimination experienced by Black women IPV survivor homeowners. Hyperpolicing may also hinder formal help-seeking behaviors from Black women IPV survivor homeowners who have concerns about being perceived as an “aggressor” and the likelihood of a dual arrest during a domestic violence call to the police [30–32]. Structural racism like racial residential segregation and gentrification have long-lasting impacts on housing stability for Black women IPV survivors; and during the pandemic, these preceding structural conditions made it more difficult for women to leave their abusive relationship.

### Relationship-level determinants

Relatedly, Black women’s descriptions of the impacts of economic abuse on their housing insecurity may reflect how IPV occurs during a crisis (e.g., pandemic). Increases in IPV during and after natural disasters can be due to housing and financial disruptions [33]. Given the widespread economic impact of the pandemic, abusive partners used economic abuse against Black women as a mechanism of control. Black women IPV survivors’ experiences of economic abuse also reflects the simultaneous sexism and SEP they bear in their relationship. Specifically, instead of obtaining work outside the home, some women were forced by their partners to stay in the home, which represents unequal power dynamics in the household (gendered domestic space) [34]. Further, economic abuse manifesting as partners defaulting on credit accounts and accumulating unpaid bills, debilitates Black women’s asset-based resources, an indicator of SEP and health [35]. Altogether, our findings indicate that economic abuse contributed to housing insecurity during the pandemic among Black women IPV survivors while also reinforcing sexism and SEP.

### Individual-level determinants

The impact of the COVID-19 pandemic on employment and wages were a central component of discussions with Black women IPV survivor renters. Black women renters described being unable to afford rent and other housing needs due to the transition from fully employed to underemployed or unemployed. The COVID-19 pandemic resulted in a high underemployment and unemployment rate among Black women [7, 8], and a wage loss elevated the risk for eviction and homelessness [36]. The economic impact of the pandemic worsens existing inequities: Black women are severely underpaid due to the intersecting effects of racism and sexism [13]. The economic vulnerabilities that Black women IPV survivors experienced during the pandemic disproportionately placed women at increased risk of future victimization and put renters, in particular, at greater risk of being caught in a cycle of homelessness and IPV.

Black women survivors in this study described their concerns about eviction, and strategies they used to maintain their housing during the pandemic. Women renting and in domestic violence shelters discussed the stress they experienced due to a potential eviction. During the pandemic, the need for domestic violence shelters was heightened, but the mandated physical distancing policies created additional barriers to meet this housing demand [37]. Therefore, current shelter residents were at risk of needing alternative housing. Black women IPV survivors also enacted “survival” strategies to maintain their housing. Specifically, survivors who were renting had to sacrifice other necessities to pay rent, while homeowners exchanged their material assets to pay their mortgage. This difference in resource allocation aligns with women’s SEP such that Black women survivors in different SEPs have varying levels of resources to navigate the stress of housing insecurity. Black women survivors without tradeable resources, utilized emotional appeals to property owners or depended on the state’s implementation of eviction moratoriums.

Some study limitations merit consideration. Purposive sampling was used to identify a sample of Black women survivors throughout the U.S.; however, women were primarily recruited from the South and based on prior or current interaction with a domestic violence agency centering Black women’s experiences. Thus, the experiences of Black women without formal interaction with an agency may be missed from this study. Study findings may not be transferable to Black women in other regions (e.g., Midwest) and residing outside of the U.S.

## Conclusions

Black women IPV survivors are facing housing insecurity within the systems of racism, sexism, and SEP; yet this group remains overlooked in the discourse on housing and IPV during the pandemic. Housing policies will continue to be ineffective for Black women IPV survivors without a deeper assessment of how sociostructural and relational dynamics uniquely impacts their experiences.

### Electronic supplementary material

Below is the link to the electronic supplementary material.

Supplementary Material 1

## Data Availability

The qualitative data generated and/or analyzed during the study are not publicly available because they contain information that could compromise participant privacy and/or consent. The corresponding author can be contacted for follow-up questions and/or concerns.

## Abbreviations

IPV: Intimate partner violence
U.S.: United States
IRB: Institutional Review Board
HIPAA: Health Insurance Portability and Accountability Act
