# Supplementary material for: Housing insecurity among black women surviving intimate partner violence during the COVID-19 pandemic: an intersectional qualitative approach
Source: BMC Public Health. 2024 Feb 16;24:501. doi: 10.1186/s12889-024-17965-5 (PMC10873942; doi:10.1186/s12889-024-17965-5)
Supplement: Supplementary file 1 — Supplementary Material 1 [file 12889_2024_17965_MOESM1_ESM.docx]

**Supplementary Table 1. Sample questions from the interview guide**

| - How would you describe what life is like for you as a Black woman in the United States? You personally, not Black women in general. |
| --- |
| - What are some of the day-to-day challenges you face as a Black woman? |
| - Overall, how has your life as a Black woman changed as a result of the COVID-19 pandemic? |
| - What is it like to manage your housing expenses while living during the COVID-19 pandemic (e.g., mortgage, rent payments, utility payments)? |
| - Since the pandemic started, can you describe a time when you were concerned about eviction? |
| - How has your interactions with your romantic partner changed since the start of the pandemic? |
| - What are some things that helped you be well as a Black woman despite some of the challenges we talked about? |
